# The role of alternative Polyadenylation in regulation of rhythmic gene expression

**DOI:** 10.1186/s12864-017-3958-1

**Published:** 2017-08-04

**Authors:** Natalia Ptitsyna, Sabri Boughorbel, Mohammed El Anbari, Andrey Ptitsyn

**Affiliations:** 1 Embry-Riddle Aeronautical University, Daytona Beach, FL 32114 USA; 2 Sidra Medical and Research Center, P.O. box 26999 Doha, Qatar; 3 Present affiliation: Gloucester Marine Genomics Institute, Gloucester, MA 01930 USA

**Keywords:** Alternative transcription, Oscillatory gene expression, Cellular circuits, Molecular diode, mathematical modeling, datamining

## Abstract

**Background:**

Alternative transcription is common in eukaryotic cells and plays important role in regulation of cellular processes. Alternative polyadenylation results from ambiguous PolyA signals in 3′ untranslated region (UTR) of a gene. Such alternative transcripts share the same coding part, but differ by a stretch of UTR that may contain important functional sites.

**Methods:**

The methodoogy of this study is based on mathematical modeling, analytical solution, and subsequent validation by datamining in multiple independent experimental data from previously published studies.

**Results:**

In this study we propose a mathematical model that describes the population dynamics of alternatively polyadenylated transcripts in conjunction with rhythmic expression such as transcription oscillation driven by circadian or metabolic oscillators. Analysis of the model shows that alternative transcripts with different turnover rates acquire a phase shift if the transcript decay rate is different. Difference in decay rate is one of the consequences of alternative polyadenylation. Phase shift can reach values equal to half the period of oscillation, which makes alternative transcripts oscillate in abundance in counter-phase to each other. Since counter-phased transcripts share the coding part, the rate of translation becomes constant. We have analyzed a few data sets collected in circadian timeline for the occurrence of transcript behavior that fits the mathematical model.

**Conclusion:**

Alternative transcripts with different turnover rate create the effect of rectifier. This “molecular diode” moderates or completely eliminates oscillation of individual transcripts and stabilizes overall protein production rate. In our observation this phenomenon is very common in different tissues in plants, mice, and humans. The occurrence of counter-phased alternative transcripts is also tissue-specific and affects functions of multiple biological pathways. Accounting for this mechanism is important for understanding the natural and engineering the synthetic cellular circuits.

**Electronic supplementary material:**

The online version of this article (doi:10.1186/s12864-017-3958-1) contains supplementary material, which is available to authorized users.

## Background

Circadian oscillation plays important role in regulation of gene expression. The number of reported cycling genes differs from study to study. Some publications report hundreds [1–3] others thousands [4] of oscillating transcripts, depending on experiment design and analysis of data. Some reports insist on majority or the entire transcriptome experiencing diurnal oscillations [5, 6]. In any case, a considerable fraction of rhythmically expressed genes is bound to modulate the activity in multiple biological pathways. Multiple other factors are known to regulate gene expression in the context of biological pathways. Alternative polyadenylation is one of such factors, sometimes considered a form of alternative splicing, but rarely mentioned in connections with circadian oscillation. Recent reviews, such as [7, 8] provide a panoramic overview of the prominent role of alternative polyadenylation in various healthy and disease states, but make no connection to the rhythmic alternations in alternative transcript population. However, some studies point specifically to the importance of such connection [9] in *Arabidopsis thaliana* and *Drosophila melanogaster* [10]. Others point at the role of polyadenylation in regulation of rhythmic protein expression [11, 12] while observing the length of PolyA tail in various transcripts in mice. In some estimations up to 35% of all alternative 3’UTR transcripts may have different turnover rate [8]. Generalizing these observations we come to a conclusion that transcription factors are not the only mechanism regulating circadian expression. Post-transcriptional mechanisms such as alternative splicing, polyadenylation, nonsense-mediated decay, etc. are also important in formation of dynamic pattern of transcripts. In this connection we would like to remember one old study reporting a perplexing pattern of alternative transcripts of suppressor of cytokine signaling (SOCS3) in mice oscillating in opposite phase to each other [13]. The paper described the occurrence of alternative microarray probes traced to alternative transcripts sharing the coding part, but resulting from alternative polyadenylation sites. This effect was first discovered in JAK-STAT (Janus Kinase - Signal Transducer and Activator of Transcription) signal transduction pathway. Counter-phased alternative transcripts were observed in brown adipose tissue, but not in white adipose or liver samples. Other elements of the same pathway such as JAK were also showing counter-phase transcripts in one tissue, but not the other. The study proposed that such pattern of alternative transcripts may represent an adaptive mechanism regulating the pathway in a tissue-specific manner by creating a constant abundance of a particular protein. For example, constant production of SOC3 from alternating short and long transcripts can block signal transduction in a particular tissue regardless of the diurnal change of the baseline. In the current study we attempt to generalize this observation and propose the model of molecular mechanism responsible for the observed pattern of counter-phase oscillation of alternative transcripts.

## Results

### Mathematical model

Let *n*_*1*_ *(t)* denote the change in abundance (for instance, relative to invariant sum of intensity of microarray control spots) of the long isoform in time and *n*_*2*_ *(t)* stands for the change abundance of the short isoform of the transcript *n* in time.

Let *r*_*p*_ describe the expression rate of the gene from which both isoforms are transcribed. Since they share the same promotor and all other functional sites except 3′ UTR polyadenylation signal, the rate is the same for both short and long transcripts. Let *p* denote the probability of transcription resulting in production of the long isoform. Then *1-p* is the probability of transcription resulting in the short isoform. The UTRs of these transcripts are different, thus we introduce separate variables for the degradation rates:

*r*_*d1*_ describes the degradation rate for the long isoform of transcript *n*_*1*_

*r*_*d2*_ describes the degradation rate for the long isoform of transcript *n*_*2*_

1 $$ \left\{\begin{array}{c}\frac{d{n}_1}{dt}=p{r}_p-{r}_{d1};\\ {}\frac{d{n}_2}{dt}=\left(1-p\right){r}_p-{r}_{d2};\end{array}\right. $$

Let’s consider the case when the baseline of expression is modulated by a simple harmonic process, such as circadian rhythm. Since the entire cell (or even the organism consisting of magnitude of cells) is modulated by the same factors, we consider the period of oscillation equal in all equations. The baseline oscillation is described by the travelling wave equation

2 $$ {r}_p=a \sin \left(\omega t+{\alpha}_1\right);{r}_{d1}=b \sin \left(\omega t+{\alpha}_2\right);{r}_{d2}=c \sin \left(\omega t+{\alpha}_3\right); $$

Here we assume that *b > c*, which means that longer transcripts have a shorter life span. This assumption models the action of miRNA that can bind the longer transcript and facilitate the decay. The shorter isoform lacks the miRNA binding site and thus can last longer, transcribing more copies of encoded protein. The overall model takes the following form:

3 $$ \left\{\begin{array}{c}\frac{d{n}_1}{dt}=pa\mathit{\sin}\left(\omega t+{\alpha}_1\right)-b\mathit{\sin}\left(\omega t+{\alpha}_2\right);\\ {}\frac{d{n}_2}{dt}=\left(1-p\right)a\mathit{\sin}\left(\omega t+{\alpha}_1\right)-c\mathit{\sin}\left(\omega t+{\alpha}_3\right);\end{array}\right. $$

The formula (14) (see Methods for complete analytic solution) allows direct calculation of the phase shift from the estimated degradation rates of short and long isoforms. These values can be estimated experimentally.

**Fig. 1:**
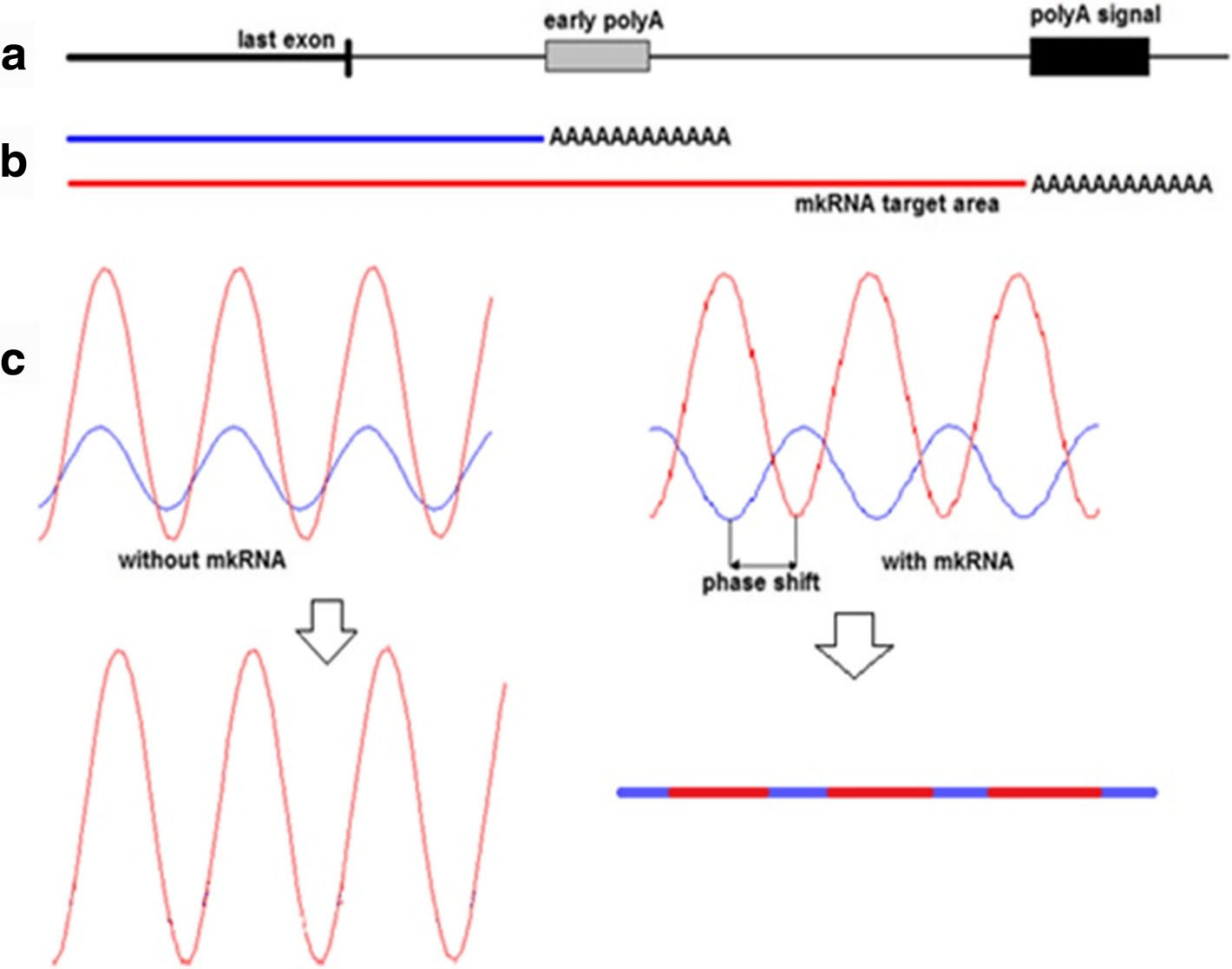
Molecular mechanism of a cellular circuit rectifier. **a** Two subpopulations of transcripts are created by occurrence of canonical distant PolyA signal and proximal non-canonical signal. **b** Transcription produces two types of mRNA that differ by a stretch of RNA that may contain functional sites such as microRNA target areas. Both transcripts share the same coding part. **c** When both transcripts have the same turnover rate, the transcript abundance has oscillating baseline. If more abundant transcript decays faster the peak abundance also shifts in time and can reach complete counter-phase (see Analytic Solution). In such case the sum of two transcripts approaches non-oscillatory steady line

Summing up isoforms *n*_*1*_ and *n*_*2*_ we can estimate the overall level of expression and amplitude of oscillation for the entire population of alternative transcripts of gene *n*. While *n*_*1*_ + *n*_*2*_ = *n* at all times, the amplitude of the resulting curve for *n* depends on the phase shift between isoforms *n*_*1*_ and *n*_*2*_. The phase lag between isoforms may have values varying between 0 and 2π. In the middle of this range, when *β*_*2*_ *−β*_*1*_ *=π* the amplitude of *n* is reduced to 0. In terms of biology, this means that gene expression oscillatory in nature at the origin can produce a constant steady production of peptides using the mechanism of differentially polyadenylated transcriptional isoforms. This mechanism provides the “power rectifier” element for the cellular circuitry. Figure 1 illustrates the action of molecular circuit rectifier. The degradation rate of mRNA which determines the turnover rate of mRNA copies and eventually the amount of synthesized protein can be affected by many factors, such as post-transcriptional modification, mRNA transport, tertiary structure, etc. However, the most well-known factor is the action of miRNA.

### Pattern datamining

Frequently referencing the same or similar data sets from independent circadian studies we could not help but notice that the pattern of alternative probe sets for the same gene showing oscillations in a different phase is quite common in different plants and animals. Here we present the results of systematic search for expression patterns indicative of counter-phase transcripts.

We present a conservative estimation of the counter-phase transcript occurrence. The standard expression microarrays are poorly suited for observation of alternative transcripts. The higher representation of 3′ UTR is usually viewed as unwanted bias that designers strive to avoid. Full length mRNAs from Refseq database are given priority over alternative shorter ESTs. Engineers also try to avoid excess probe sets interrogating the same gene in order to make quantitative estimation of gene expression more consistent. As a result we are only able to observe alternative polyadenylation through unintended imperfection on microarray design.

The phase estimation procedure described previously provides for each probe an estimation of the phase among one of six phase classes discretized by cyclic shift *π i /6, (i = 1..6)* and a corresponding *p*-value for each estimate. The *p*-value calculation is obtained from the bootstrap analysis described in Algorithm 1. The latter can be used to filter probes with low statistical confidence on their phase estimate. We used the mouse annotation data (available from Affymetrix and on the shared github source code) to identify multiple probe sets interrogating expression of the same gene. All probes that correspond to the same gene symbol are gathered in a same probe set. The next step of the analysis is to generate phase differences within each probe set. All probe pairs in each probe set are used to compute the absolute value of phase difference.

**Fig. 2:**
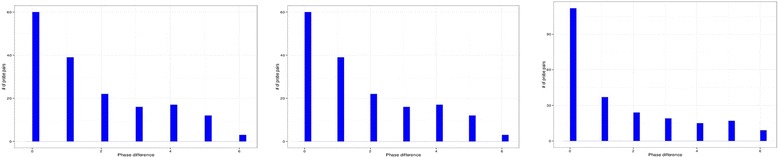
Distribution of the number of probes as function of phase difference for three mouse tissues (*from left to right*: white adipose tissue, brown adipose tissue, liver). In all three tissues there are many genes with multiple probe sets oscillating in a different phase. Moreover, there is a pronounced peak corresponding to probes oscillating in opposite phases

Figure 2 shows the distribution of phase differences for three mouse tissues. We used a threshold *p*-value of 0.1 to filter probes with very low confidence on phase estimation. There is a peak around the zero phases for the different tissues. This result is expected since the probes are designed to provide a robust estimation of expression levels. Probe sets and separate probes within each set reporting results inconsistent with other probes and probe sets for the same gene tend to be eliminated from the chip on early design stages. As a result, the majority of alternative probe sets report abundance of the same transcript and shows no phase difference. There is a degree of uncertainty in identification of phase, considering the low sampling rate and high level of technical variation in microarray data. Thus, we expect high number of alternative probesets with phase difference by one time point (second bar on the diagrams in Fig. 2). Likewise, there should be progressively fewer alternative probesets with larger phase difference. However, the diagrams show pronounced peaks corresponding to approximately opposite phases of oscillation.

**Fig. 3:**
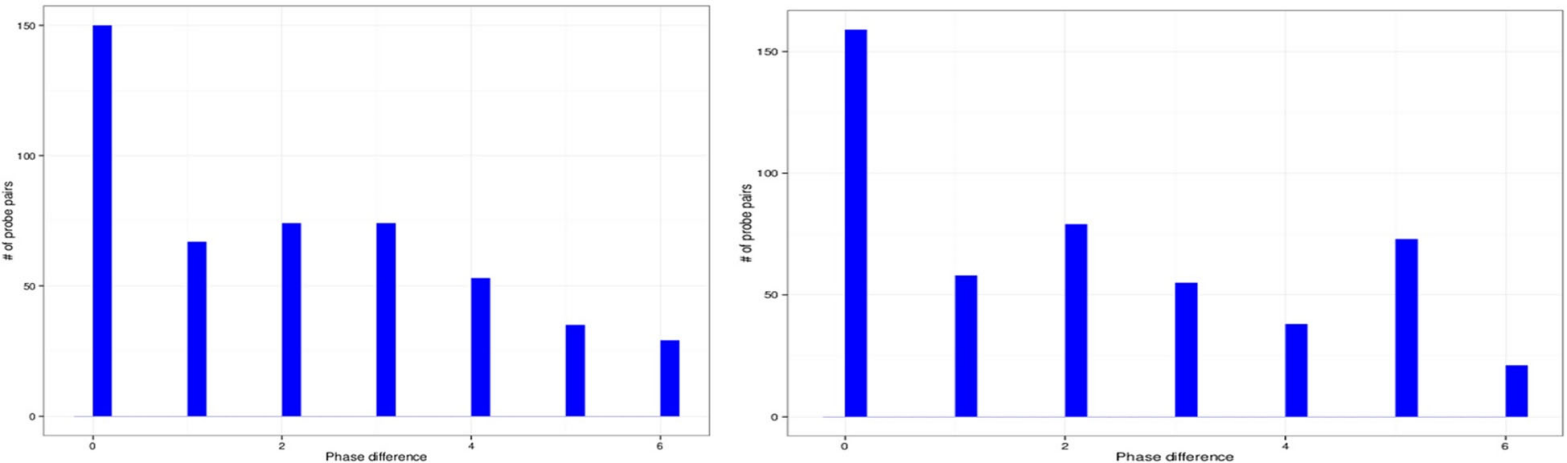
Distribution of the number of probes as a function of phase difference for *Arabidopsis thaliana.* The results are similar for both data sets from the University of Warwick [14] (*left figure*) and from UC Davis [13] (*right figure*). In both data the highest bar corresponds to pairs of alternative probesets that oscillate as expected with no phase difference. However, in both cases the second largest number of probe pairs oscillates with a significant phase difference

We tested the significance of these visible bumps on diagrams in Fig. 2. Let’s assume that in ideal microarray design all probe sets report consistent abundance of transcripts and there is no phase difference between alternative probe sets. In this case the first bar on Fig. 2 would be dominating, but we would still observe other bars due to technical variation and uncertainty in peak time estimations. However, if perceived phase difference was caused by stochastic variation only, we would observe progressively fewer cases with larger phase difference. It is expected that as the phase difference increases the count would decrease in an exponential manner. Therefore we can test the hypothesis that the observed distribution of phase difference has some stochastic decay. In order to test this hypothesis we fitted the phase difference distribution by a Poisson distribution. The advantage of using a Poisson distribution is that it can capture random variables that have stochastic decays. In addition since we have a discrete number of bins for phase difference, Poisson distribution is a good choice for discrete support. After fitting the distribution to the phase difference data, we applied a Chi-Square test to verify if this fitting is statistically valid G. Table 1 summarizes the Poisson distribution fitting and the hypothesis testing results. We observe that, in the five datasets, the null hypothesis can be rejected and that the stochastic decay does not completely explain the phase difference distribution. We are aware that Poisson distribution does not capture all possible distributions with stochastic decay. We also performed non-parametric estimation of the phase difference distribution. The results are consistent with the tests in Table 1 (data not shown, see Additional file 1: Supplemental method). The distributions in Figs. 2 and 3 cannot be explained by stochastic variation and reflects a fraction of probes that oscillate consistently in opposite or near-opposite phase to each other. This observation is also true for all analyzed datasets (see complete list of probe sets with phase differences in Additional files 2 and 3).

**Table 1:** Phase difference distribution and stochastic decay hypothesis testing

Dataset	Lambda	X-squared	*p*-value
Brown adipose tissue	1.64	74.21	5.5e-14
White adipose tissue	1.5	46.45	2.4e-08
Liver	1.46	267.06	2.2e-16
Arabidopsis (UC Davis)	2.12	403.19	2.2e-16
Arabidopsis (Warwick)	2.07	272.88	2.2e-16

Lambda denotes the parameter of the Poisson distribution, X-square and *p*-value are the X2 hypothesis testing result. The Null hypothesis is that the phase difference distribution follows a Poisson distribution

We applied the same analysis to *Arabidopsis thaliana* circadian gene expression data. The data comes from the published sources (GEO GSE8365, GSE5612) and the primary analysis has been previously published [14, 15] and later re-analyzed and published again [16]. Additional challenges in identifying alternative probesets in *Arabidopsis thaliana* microarray come from the less extensive annotation (also available from Affymetrix). For this analysis we considered probesets representing the same gene if any of the following fields were identical: RefSeq Transcript ID, AGI and Entrez Gene. Figure 3 shows that overall the phase difference distribution is similar to the analysis based on mouse data with some differences in the distribution shape. The occurrence of alternative transcripts oscillating with pronounced phase difference in such distant organism leads to conclusion that the mechanism creating phenomena is likely to be common for all eukaryotic organisms.

## Discussion

Regardless of the source of oscillation, the rhythmic nature of expression demands a significant revision of the way we understand and model the function and regulation of genes. One of the previously published models predicted that for a rhythmically expressed gene addition of miRNA may have two different effects: either expected decrease or surprising increase of transcript abundance, depending on timing of miRNA action [17]. In case of alternative polyadenylation, we have first noted, investigated and reported a strange abnormality in expression of alternative probe sets reporting activity of the same genes [13]. Disagreement in intensity among alternative probe sets is usually attributed to cross-hybridization, flaws in microarray design or manufacturing or other factors reflecting technical rather than biological variation. Indeed, experiments comparing only single points in time are insufficient to explain such discordance. Observation of complete circadian (or other periodic) time leaves no doubt that at least some of the alternative probe sets report biologically relevant rather than technical variation.

Our model shows that such strange behavior of alternative probes is not only natural; they are performing an important function. This function eliminates the effect of oscillation in transcription mechanism. Other studies have already reported pervasive oscillation of the entire transcriptome (see [18, 19, 20] for review). Current study presents the mechanism for rectification of constant baseline oscillation. If there is such mechanism than the default state of gene expression must be rhythmic. Our observations show that this mechanism is common in among plant, mouse and human genes. However, our study tends to underestimate the occurrence of alternative transcripts. On the chips used to produce this data thousands of genes are interrogated by a single probe set only. In cases when original CEL files are available for Affymetrix microarrays it is possible to analyze more genes with alternative transcripts by low level single probe analysis. However, individual probes may not be uniformly distributed to represent all transcripts and are less reliable in quantitative estimation of transcript abundance. The true occurrence of such mechanism is yet to be determined in a specially designed experimental study using a different detection mechanism.

Most of approaches in bioengineering and synthetic biology make no account for oscillation [21]. We believe a significant advancement in Pathway Engineering will require better understanding of the principles on which complex biological systems are organized. One of the principles is oscillation in production of cellular components, signal transduction and energy metabolism. Ignoring the fact of oscillation is only possible on the early steps or when implementing most primitive constructions. This study offers one of the components for building artificial cellular systems or re-engineering the existing cells based on the knowledge of the rhythmic nature of gene expression. This component is a functional analog of a diode in electronic circuits and can rectify oscillations occurring in biological circuits due to the oscillatory nature of gene expression.

## Conclusion

The findings described in this paper may have practical applications in pathway engineering and synthetic biology. Our model provides the mechanism for re-engineering of existing biological pathways in a living cell or de novo design of cellular circuits. The model predicts that it is possible to find the parameters (such as miRNA site with certain affinity) regulating the ratio of alternatively adenylated transcripts. Manipulating such ration allows changing the amplitude of a particular gene expression or even complete elimination of oscillation. Constant abundance of a gene product can be used for production purpose to maximize the output of a peptide or an enzyme producing the product of interest. Alternatively this mechanism can be engineered to block unwanted pathways such as apoptosis or cell motivity, etc., or to keep certain pathways active at any time. Likewise, the same model can be used to create a blueprint for constructing artificial genes with certain properties. For example, the formula given in the description can be used to select parameters of the artificial gene (affinity of early and late PolyA sites, affinity of microRNA binding site) in order to create the desirable amplitude of oscillating product abundance.

## Methods

### Data sources

The mouse gene expression profiles was obtained in the original study of circadian gene expression in adipose tissues [22]. The AKR/J mice acclimated to a 12 h light: 12 h dark cycle, were harvested in sets of 3–5 mice at 4 h intervals in duplicates over a 24 h period. Total RNA samples from inguinal (iWAT) white adipose tissue, brown adipose tissue (BAT), and liver have been assayed by Affymetrix U74 GeneChip microarrays.

#### The plant (*Arabidopsis thaliana*) data sets

We used two independent data sets similar in experiment design [14, 15]. Seedlings were entrained in 12-h white light (light source was cool white fluorescence tubes)/12-h dark cycles for 7 days before being released into free-running conditions of continuous white light at 22 °C. Starting at subjective dawn of day 8 [14] or day 9 [15], tissue was harvested every 4 h over the course of the next 44 h. Following standard protocols labelled cRNA targets were prepared from total RNA and hybridized to Affymetrix Arabidopsis expression GeneChips according to the manufacturer’s instructions.

#### Analytic solution

We integrate each equation separately with respect to time. The solution of the system is:

4 $$ \left\{\begin{array}{c}{n}_1(t)=-\frac{pa}{\omega}\mathit{\cos}\left(\omega t+{\alpha}_1\right)+\frac{b}{\omega}\mathit{\cos}\left(\omega t+{\alpha}_2\right);\\ {}{n}_2(t)=-\left(1-p\right)\frac{a}{\omega}\mathit{\cos}\left(\omega t+{\alpha}_1\right)+\frac{c}{\omega}\mathit{\cos}\left(\omega t+{\alpha}_3\right);\end{array}\right. $$

The rotating-vector description of simple harmonic oscillation provides a neat way of rewriting *n*_1_ and *n*_2_ as single harmonic oscillations:

5 $$ {n}_1(t)=A\mathit{\cos}\left(\omega t+{\beta}_1\right); $$

6 $$ {n}_2(t)=B\mathit{\cos}\left(\omega t+{\beta}_2\right); $$

**Fig. 4:**
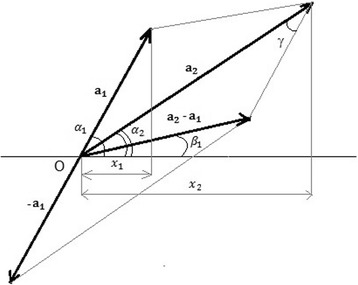
Geometric solution for the phase shift between oscillating transcript isoforms. See detailed description in the text

The following geometric solution is illustrated in Fig. 4. The harmonic oscillations *x*_1_ and *x*_2_ can be represented as two vectors a_1_ and a_2_ that rotate around their tails, which are pivoted at the origin *O*. The angular speed of the rotation is equal to *ω*. As the vectors rotate around the origin their projections *x*_1_ and *x*_2_ on the horizontal axis vary cosinusoidally. Hence we have

7 $$ \gamma =\frac{2\pi -2\Big(\pi -{\alpha}_1+{\alpha}_{2\Big)}}{2}={\alpha}_1-{\alpha}_2; $$

8 $$ {A}^2={\left|{\mathrm{a}}_1\right|}^2+{\left|{\mathrm{a}}_2\right|}^2-2\mid {\mathrm{a}}_1\mid \mid {\mathrm{a}}_2\mid \mathit{\cos}\left({\alpha}_1-{\alpha}_2\right); $$

9 $$ {A}^2={\left(\frac{pa}{\omega}\right)}^2+{\left(\frac{b}{\omega}\right)}^2-\frac{2pab}{\omega^2}\mathit{\cos}\left({\alpha}_1-{\alpha}_2\right); $$

Therefore *n*_1_(*t*) = *A* cos(*ωt* + *β*_1_), with

10 $$ A=\sqrt{{\left(\frac{pa}{\omega}\right)}^2+{\left(\frac{b}{\omega}\right)}^2-\frac{2pab}{\omega^2}\mathit{\cos}\left({\alpha}_1-{\alpha}_2\right);} $$

11 $$ \mathit{\tan}{\beta}_1=\frac{\mid {a}_2\mid \mathit{\sin}{\alpha}_2-\mid {a}_1\mid \mathit{\sin}{\alpha}_1}{\mid {a}_2\mid \mathit{\cos}{\alpha}_2-\mid {a}_1\mid \mathit{\cos}{\alpha}_1}=\frac{b\mathit{\sin}{\alpha}_2-pa\mathit{\sin}{\alpha}_1}{b\mathit{\cos}{\alpha}_2-pa\mathit{\cos}{\alpha}_1}; $$

Similarly, we can get the expression for *n*_2_:

$$ {n}_2(t)=B\mathit{\cos}\left(\omega t+{\beta}_2\right); $$

where.

12 $$ B=\sqrt{{\left(1-p\right)}^2{\left(\frac{a}{\omega}\right)}^2+{\left(\frac{c}{\omega}\right)}^2-\frac{2\left(1-p\right)ac}{\omega^2}\mathit{\cos}\left({\alpha}_1-{\alpha}_3\right);} $$

13 $$ \mathit{\tan}{\beta}_2=\frac{c\mathit{\sin}{\alpha}_3-\left(1-p\right)a\mathit{\sin}{\alpha}_1}{c\mathit{\cos}{\alpha}_3-\left(1-p\right)a\mathit{\cos}{\alpha}_1}; $$

And the phase difference is.

14 $$ {\beta}_2-{\beta}_1=\mathit{\arctan}\left(\frac{c\mathit{\sin}{\alpha}_3-\left(1-p\right)a\mathit{\sin}{\alpha}_1}{c\mathit{\cos}{\alpha}_3-\left(1-p\right)a\mathit{\cos}{\alpha}_1}\right)-\mathit{\arctan}\left(\frac{b\mathit{\sin}{\alpha}_2-pa\mathit{\sin}{\alpha}_1}{b\mathit{\cos}{\alpha}_2-pa\mathit{\cos}{\alpha}_1}\right). $$

#### Phase assignment and phase confidence algorithm

The algorithm used in the analysis of the data is based on resampling techniques. Indeed, we use the maximum entropy bootstrap algorithm to generate a large number of replications of a given gene expression time series. Then, we calculate a bootstrapped *p*-value to test for circadian genes, and finally we construct a bootstrap percentile confidence interval that will be used to assign a phase to each oscillating gene.
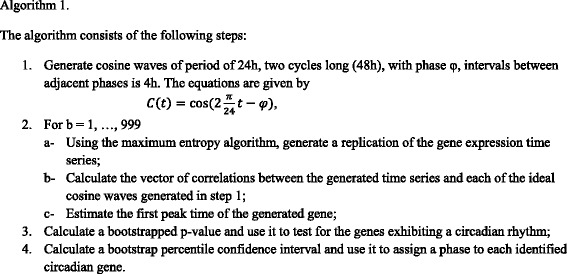


The complete description with source code and test results has been published in [23].

## Additional files

Additional file 1: Supplemental method. In addition to Chi-Square test summarized in Table 1 we run a Monte Carlo simulation with 1000 repetitions with the option simulate.p.value as described in https://stat.ethz.ch/R-manual/R-devel/library/stats/html/chisq.test.html. This supplemental file provides description and *p*-values obtained in simulations. (DOCX 10 kb)

Additional file 2: Supplemental Data Tables. This zip archive contains the results of analysis of phase difference among redundant probe sets in mouse liver, mouse brown fat, mouse white fat and two independent studies of *Arabidopsis thaliana* timeline gene expression. (ZIP 32 kb)

Additional file 3: Supplemental Initial Data Tables. This zip archive contains the initial timeline data for probe sets in mouse liver, mouse brown fat, mouse white fat originally published in Zvonic et al. [21]. (ZIP 3710 kb)

## Abbreviations

AGI: Arabidopsis Genome Initiative
BAT: Brown adipose tissue
cRNA: Antisense amplified RNA
GEO: Gene Expression Omnibus
iWAT: Inguinal white adipose tissue
JAK: Janus Kinase
miRNA: micro Ribonucleic Acid
mRNA: Messenger Ribonucleic Acid
SOCS3: Suppressor of cytokine signaling
STAT: Signal Transducer and Activator of Transcription
UTR: Untranslated region
