# Supplementary material for: The role of alternative Polyadenylation in regulation of rhythmic gene expression
Source: BMC Genomics. 2017 Aug 4;18:576. doi: 10.1186/s12864-017-3958-1 (PMC5544998; doi:10.1186/s12864-017-3958-1)
Supplement: Supplementary file 1 — Supplemental method. In addition to Chi-Square test summarized in Table 1 we run a Monte Carlo simulation with 1000 repetitions with the option simulate.p.value as described in https://stat.ethz.ch/R-manual/R-devel/library/stats/html/chisq.test.html. This supplemental file provides description and p-values obtained in simulations. (DOCX 10 kb) [file 12864_2017_3958_MOESM1_ESM.docx]

By design of the micro-array chips it is expected that zero phase difference should be dominant in the count of the number of probes within a gene with a given phase difference. Beyond zero phase difference it is also expected that as the phase difference increases the count would decrease in an exponential manner. Therefore the hypothesis here is that the ideal distribution of phase difference has some stochastic decay. This is of course an idealized scenario from the engineering design of the chip. In order to test this hypothesis we fitted the phase difference distribution by a Poisson distribution then we applied a Chi-Square test to verify if this hypothesis is correct. Table A1 summarizes the Poisson distribution fitting and the hypothesis testing results. We observe that, in the five datasets, the null hypothesis can be rejected and that the stochastic decay does not completely explain the phase difference distribution. We are aware that Poisson distribution does not capture all possible distributions with stochastic decays and hence we analyzed in the following paragraph the use of non-parametric estimation of the phase difference distribution.

**Table A1.** *Phase difference distribution and stochastic decay hypothesis testing. Lamda denotes the parameter of the Poisson distribution, X-square and p-value are the X2 hypothesis testing result. The Null hypothesis is that the phase difference distribution follows a Poisson distribution.*

| dataset | lambda | X-squared | p-value | P-value (Monte Carlo) |
| --- | --- | --- | --- | --- |
| Brown adipose tissue | 1.64 | 74.21 | 5.5e-14 | 0.000999 |
| White adipose tissue | 1.5 | 46.45 | 2.4e-08 | 0.000999 |
| Liver | 1.46 | 267.06 | 2.2e-16 | 0.000999 |
| Arabidopsis (UC Davis) | 2.12 | 403.19 | 2.2e-16 | 0.000999 |
| Arabidopsis (Wisconsin) | 2.07 | 272.88 | 2.2e-16 | 0.000999 |

For each tissue, let’s consider the probes with a p-value < 0.1. Form classes of probes corresponding to each gene. For each class, calculate the pairwise phase difference and keep only those with an absolute value > = 2. Define X as the phase difference absolute value. X is a discrete random variable taking values in {0, 1, 2, 3, 4, 5, and 6}. Its probability function is summarized as follows:

1. Brown adipose tissue data set

| x | 0 | 1 | 2 | 3 | 4 | 5 | 6 |
| --- | --- | --- | --- | --- | --- | --- | --- |
| P({X=x}) | 60/169 | 39/169 | 22/169 | 16/169 | 17/169 | 12/169 | 3/169 |

1. White adipose tissue data set

| x | 0 | 1 | 2 | 3 | 4 | 5 | 6 |
| --- | --- | --- | --- | --- | --- | --- | --- |
| P({X=x}) | 56/141 | 23/141 | 21/141 | 24/141 | 12/141 | 4/141 | 1/141 |

1. Liver data set

| x | 0 | 1 | 2 | 3 | 4 | 5 | 6 |
| --- | --- | --- | --- | --- | --- | --- | --- |
| P({X=x}) | 112/233 | 37/233 | 24/233 | 19/233 | 15/233 | 17/233 | 9/233 |

4- Arabidopsis (UC Davis)

abs_dif

0 1 2 3 4 5 6

159 58 79 55 38 73 21

> sum(abs_dif)

[1] 1024

5- Arabidopsis (Wisconsin)

abs_dif

0 1 2 3 4 5 6

150 67 74 74 53 35 29

> sum(abs_dif)

[1] 998

From the three tables above, we can observe that the shape of the probability function doesn’t have a decay shape. It has a decreasing trend but then it increases, and it decreases again. This observation is also true for all analyzed datasets.
